# Immunoreactivity of the 14F7 Mab (Raised against N-Glycolyl GM3 Ganglioside) as a Positive Prognostic Factor in Non-Small-Cell Lung Cancer

**DOI:** 10.1155/2012/235418

**Published:** 2012-02-26

**Authors:** Rancés Blanco, Charles E. Rengifo, Mercedes Cedeño, Milagros Frómeta, Enrique Rengifo, Adriana Carr

**Affiliations:** ^1^Laboratory of Recognition and Biological Activity Assays, Department of Quality Control, Center of Molecular Immunology, Playa, P.O. Box 16040, Havana 11600, Cuba; ^2^Department of Pathology, Manuel Fajardo General Hospital, Plaza de la Revolución, Havana 10400, Cuba; ^3^Research and Development Direction, Center of Molecular Immunology, Havana, Cuba

## Abstract

Lung carcinoma is the leading cause of cancer-related mortality worldwide. Therefore, numerous studies are focusing on the assessment of other biological and molecular prognostic factors in these tumors. We evaluated the relationship between 14F7 Mab reactivity, pathological features, DNA-content and S-phase fraction (SPF), and their impact in the survival of NSCLC patients. Hematoxylin and eosin staining and immunohistochemistry optical microscopy assays as well as DNA content and SPF measuring using flow cytometry were performed. The 14F7 reactivity was widely observed in NSCLC sections, no depending of the clinicopathological characteristics. We also obtained differences in the intensity of reaction with 14F7 as well as in the SPF between diploid and aneuploid carcinomas. Patients with diploid tumors showing higher SPF and 14F7 reaction joint to a low mitotic index displayed higher survival rates. Our results are in agreement with the assumption of the possible positive prognostic value of 14F7 staining in NSCLC.

## 1. Introduction

Malignant neoplasms of respiratory system are one of the most common human cancers. Among them, the malignancies of lung have a very poor prognosis, representing the leading cause of cancer-related mortality worldwide [1]. There are two main variants of the disease, non-small-cell lung cancer (NSCLC) and small cell lung cancer (SCLC). NSCLC is the most common form of the disease, accounting for approximately 85% of all cases [2]. Despite of the recent advances in cancer therapy, the therapeutic option available for patients with disease that cannot be surgically managed has traditionally been limited to chemotherapy, providing a modest survival benefit [3]. 

Nowadays, research efforts are focusing on the better understanding of tumor biology and genetics of lung tumors in order to select better molecules as target, leading to more effective treatments for this often difficult disease [3]. Among these molecules, gangliosides have been included [4]. Gangliosides are sialic-acid-containing glycosphingolipids engaged in many biological events that take place at vertebrate's cell membrane [5]. Usually, malignant cells expressing aberrant glycolylated pattern in the gangliosides composition have been identified by immunohistochemistry.

It is known that N-acetylneuraminic acid (NeuAc) is the most abundant sialic acid form expressed in humans. In contrast to NeuAc, the expression of NeuGc (N-glycolylneuraminic acid) forming the structure of gangliosides and/or other glycoconjugates (Hanganutziu-Deicher antigen) has been considered as a tumor-associated antigen [6]. The aberrant expression of the NeuGc acid residues has been considered to be related with the altered metabolism of malignant cells [7–9]. Normal human cells are incapable of synthesizing NeuGc due to a specific inactivating mutation in the cytidine monophospho-N-acetylneuraminic acid hydroxylase (CMP-NeuAc hydroxylase) gene [10]. The expression of N-glycolyl-containing gangliosides has been found in a variety of human malignancies as compared with normal tissues, with these molecules becoming attractive targets for cancer immunotherapy [11, 12].

Recently, van Cruijsen et al. published the expression of N-glycolyl GM3 ganglioside (NeuGcGM3) in non-small-cell lung cancer using tissue micro array analysis and the 14F7 Mab [13]. 14F7 is the first IgG1 highly specific against NeuGcGM3 reported in the literature [11]. The present study was undertaken to evaluate the relationship between 14F7 Mab reactivity, some pathological features, tumour cell proliferation (S-phase fraction) and DNA content (ploidy) in NSCLC. We also assessed the prognostic significance of 14F7 Mab staining in these patients. In addition, samples of nasopharyngeal carcinoma as well as normal and nontumoral tissues sections were included in this study.

## 2. Materials and Methods

We used the 14F7 Mab, a murine IgG1 highly specific against the variant N-glycolylated of GM3 ganglioside. 14F7 Mab was produced by the Center of Molecular Immunology, Havana, Cuba as it was previously described [11].

### 2.1. Tissue Specimens and Previous Processing

A number of 14 and 32 routinely processed, formalin-fixed and paraffin-embedded archival samples with diagnosis of nasopharyngeal carcinoma and lung cancer, respectively, as well as 3 cases of nontumoral entities of nasopharynx and 4 of normal human lung were taken from both the Pathology Department of Manuel Fajardo General Hospital and the Tumor Bank of the Center of Molecular Immunology, after obtaining informed consent and the approval consent by the institutional ethical committees. Five micrometer serial sections from each block were obtained in a micrometer (Leitz 1512, Germany) and mounted on plus slides (Dako S2024, Carpinteria, USA). All sections were attached to the slide by heating in a 70°C oven for 1 h. Afterward the slides were kept at room temperature until they were used.

The slides were dewaxed in xylene and rehydrated in graded ethanol series as usually and endogenous peroxidase activity was blocked with dual endogenous enzyme block solution (Dako S2003, Carpinteria, USA) for 10 minutes. All sections were washed in distilled water for 10 minutes and rinsed with washing buffer (Dako K1494, Carpinteria, USA).

### 2.2. Immunohistochemical Staining

Subsequently, slides were placed in a humid chamber and incubated with the primary mouse anti-NeuGcGM3 ganglioside 14F7 Mab for 1 h at room temperature. Negative controls were performed substituting primary antibody for washing buffer and sections of colonic adenocarcinoma were taken as positive control [14].

After two rinses in washing solution the slides were incubated with a polymer/HRP (Dako E0354, Carpinteria, USA) for 30 minutes each one. Between incubations, slides were washed for 10 minutes. Afterward, enzymatic activity was visualized with DAB substrate chromogen solution (Dako K3465, Carpinteria, USA) and the tissues were counterstained with Mayer's Hematoxylin (Dako S2020, Carpinteria, USA). The samples were dehydrated and mounted with a synthetic medium (Sakura 1467, Finetek Europe B.V).

### 2.3. Immunohistochemical Evaluation

The intensity of reaction of each samples was judged as negative (−), weak (+), moderate (++), and strong (+++), and combinations of these patterns were used to express intermediate levels of immunostaining. Additionally, the most representative regions of each section were selected and the percentage of tumor cells showing 14F7 Mab staining in them was estimated using the 10x objective lens. It was classified as 0 (negative to less than 5%), 1+ (6–25%), 2+ (26–50%), and 3+ (more than 50%). The results in agreement with two observers were considered as final.

### 2.4. Pathological Features Evaluation

Tumor sections were stained with hematoxylin and eosin (HE). Morphologic parameters such as histopathological classification, grade of differentiation, degree of cellular pleomorphism, and mitotic and necrosis indexes were evaluated for an expert pathologist (Charles E. Rengifo) in each tumor tissues.

The cytomorphologic characteristics such as cell and nuclear size, cellular shape, chromatin pattern, nucleoli, and amount of cytoplasm was evaluated to express the degree of cell pleomorphism and was scored as follows: 0 (no evident cell pleomorphism) 1, (low), 2 (moderate), and 3 (high) cell pleomorphism. Mitotic index was recorded qualitatively and was expressed as was previously described for degree of cell pleomorphism. The degree of tumor necrosis (necrosis index) on the section was determinated using a low-power field (10x objective lens) and was scored subjectively as follows: 0 (no necrosis), 1 (less than 50% of necrosis areas per field), and 2 (more than 50% of necrosis areas per field).

### 2.5. Flow Cytometric DNA-Content Measurement

The method as was previously described [15] with some modifications was performed. Briefly, we evaluated the DNA content (ploidy) and the proliferative characteristics (percentage of cells in S-phase) by the application of flow cytometry methodology (FCM) using nuclei isolated from formalin-fixed and paraffin-embedded tissues.

When possible, samples with no or minimal tumor necrosis were studied. Two or three 50 *μ*m sections were obtained from each tissue block. Afterward, the samples were dewaxed in xylene, rehydrated in a series of progressively decreasing concentrations of ethanol and finally washed in distilled water. The tissues were treated with 0.2% citrate acid pH6 solution for 2 h at 80°C. Single-cell suspensions were then prepared by incubation at 37°C for 10 minutes in a 0.5% pepsin solution (Sigma Chemical Co., St. Louis, MO) (pH 1.5). After the enzymatic digestion by pepsin, disaggregation was completed mechanically. Disaggregated material was filtered through 50 *μ*m nylon mesh and routinely washed. Dissociated nuclei were adjusted to a final concentration of 1 × l0^6^ nuclei/mL and were then stained with propidium iodide at a final concentration of 40 *μ*m/mL (Sigma Chemical Co., St. Louis, MO). The samples were analyzed on an Ortho Cytoron Absolute flow cytometer (Ortho Diagnostic Systems, Tokyo, Japan) equipped with a 15 MW argon laser. An average of 10.000 nuclei was evaluated for each sample, at a low flow rate. Afterward, the data obtained were analyzed using ModFit LT software (DNA Modeling System) version 2.0 (Verity Software House, Inc.). A sample was considered DNA diploid if, on the histogram, there was a single peak in the G_0_/G_1_ phase and DNA aneuploid, if there was at least one distant G_0_/G_1_ population. Tetraploid as well as multiploid histograms were classified into the aneuploid group.

### 2.6. Statistical Analysis

Statistical analysis was performed with nonparametric tests, since the distribution of 14F7 Mab reactivity in NSCLC was nonnormal. The correlation between pathological variables and the intensity of reaction with 14F7 Mab as well as the percentages of positive cells was assessed by Spearman ranks correlation coefficients. Chi-square test was used to compare dichotomic variables. Survival distribution was estimated by the Kaplan-Meier method. Survival comparison was performed by two-sided log-rank tests. The criterion for statistical significance was *P* < 0.05.

## 3. Results

### 3.1. Patient Description and Pathological Features

Table 1 showed a summary of patient characteristics and some pathological features. The median of patient age at presentation was 55 years (ranged from 41 to 74 years). Median overall survival of the population was 64.06 months (ranged from 3 to 91.29 months).

### 3.2. Higher Levels of 14F7 Mab Reactivity Were Detected in Non-Small-Cell Lung Cancer Sections

**Figure 1:**
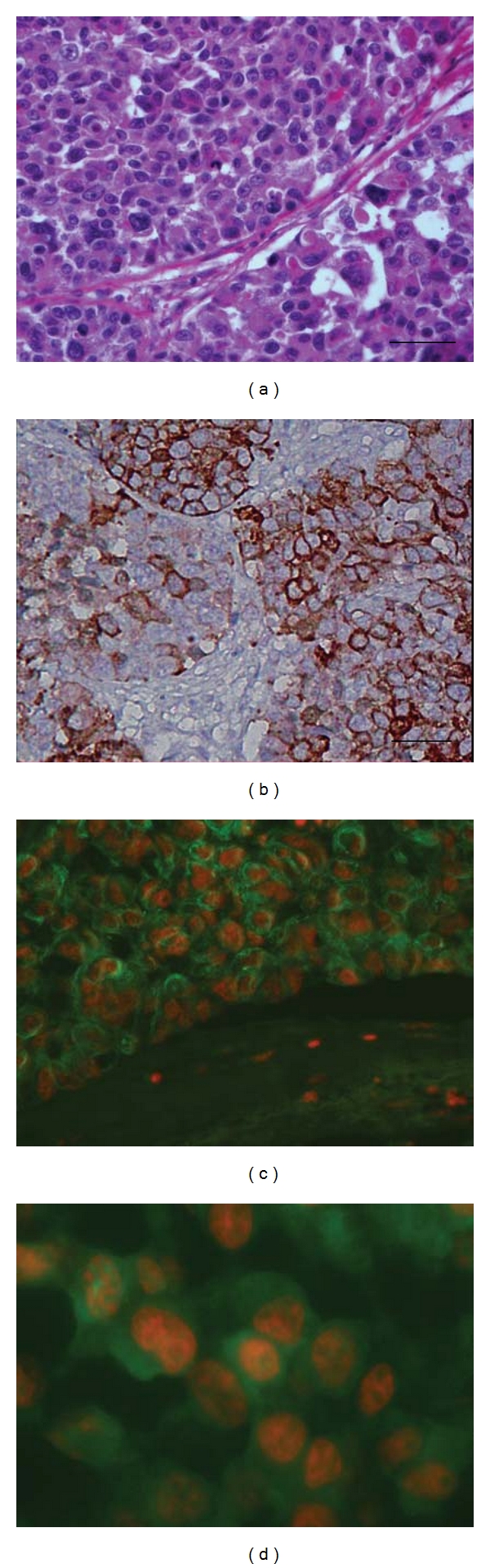
Microphotographs of poorly differentiated adenocarcinoma of the lung. (a) Hematoxylin and eosin coloration. Immunohistochemical localization of cytokeratin was performed to verify the quality of the formalin-fixed and paraffin-embedded tissues. (b) Conventional immunoperoxidase techique counterstaining with Mayer's Hematoxylin. Black bar = 50 *μ*m. (c) and (d) Conventional fluorescent and confocal microscopy, respectively. FITC-labeled cytokeratin (green signal) was used. Nuclei were contrasted with propidium iodide (red signal) (40 and 100x magnification, resp.).

Firstly, we evaluated the immunohistochemical localization of cytokeratin in order to verify the quality of the formalin-fixed and paraffin-embedded tissues. The samples remained well preserved, conserving intact the molecular antigenic determinants and showing a good morphology (Figure 1). 

**Figure 2:**
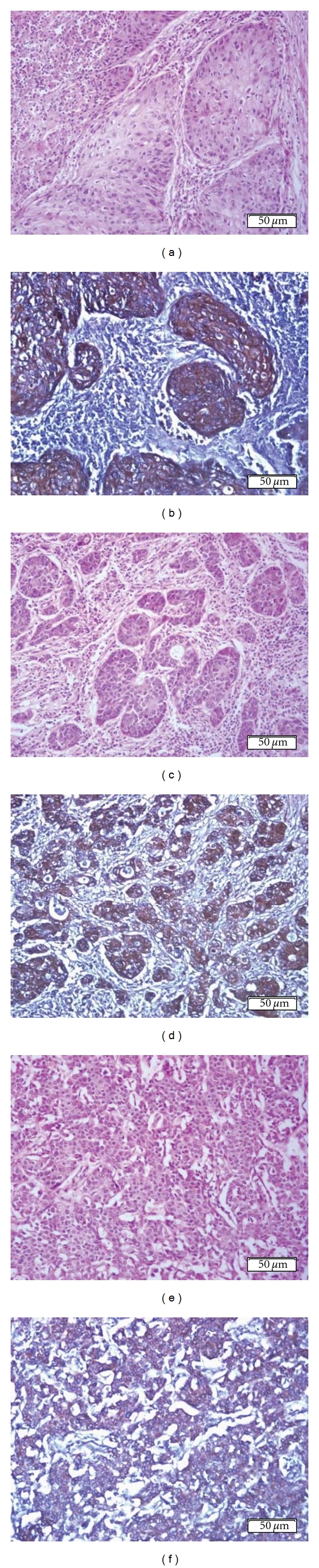
Hematoxylin and eosin staining of squamous (a), adenosquamous (c), and large cell carcinoma of the lung (f). See, the intense reaction of 14F7 Mab mainly located in cell membrane and also in the cytoplasm of malignant epithelial cells of squamous (b) and adenosquamous carcinomas (d). A moderate immunostaining with 14F7 Mab is showed in large cell carcinoma (f). Black bar = 50 *μ*m.

No staining with 14F7 Mab was observed in normal lung sections (0/4). However, the reactivity of 14F7 Mab was evidenced in 29/31 (93.5%) of lung cancer samples not taking into account the type of carcinoma (Table 2). The intensity of reaction of 14F7 Mab varied from weak to intense, but 25/29 (86.2%) of lung carcinomas showed a moderate-to-intense recognition. On the other hand, more than 25% of malignant cells were reactive with 14F7 Mab in 27/29 (93.1%) of tumors. The pattern of staining of 14F7 Mab was finely granular and was located on both cell membrane and cytoplasm of malignant cells, although the cytoplasmatic pattern was mainly detected (Figure 2). No nuclear reactivity with 14F7 Mab was evidenced. 

### 3.3. The 14F7 Mab Recognition Was Not Related with the Pathological Features of Patients

No correlation was observed between the intensity of 14F7 Mab reaction and the percentage of positive cell with the distribution of gender, age, tumor size, tumor stage, histopathological type, grade of differentiation, nor necrosis index. Interestingly, a noticeable correlation was detected between the percentage of 14F7 Mab positive cells and the degree of cell pleomorphism (*P* = 0.0001), but not with the intensity of reaction (*P* = 0.0713) (Table 3). 

### 3.4. DNA Content, Mitotic Index and Diploid S-Phase Fraction Correlate with the 14F7 Mab Staining

**Figure 3:**
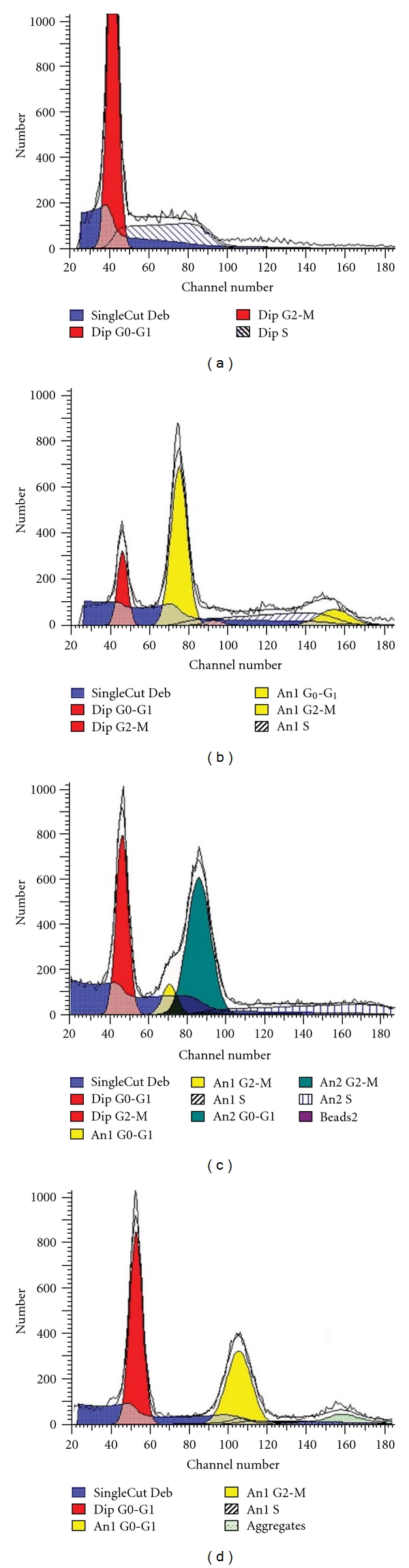
DNA-content (ploidy) analysis in non-small-cell lung carcinoma samples measured by the flow cytometric profile and analyzed by Modfit LT software. Cells prepared by the Hedley method [15] and stained with propidium iodide. DNA histograms of diploid (a), aneuploid (b), multiploid (c), and tetraploid (d) tumors.

Flow cytometry analysis for DNA content was performed in 20/32 (62.5%) non-small-cell lung cancer samples. Eleven (55.0%) were diploid and 9 (45.0%) aneuploid. The median coefficient of variation (CV) was 7.4 (range 3.9–13.89) (Figure 3). A significant correlation was detected between the intensity of reaction of 14F7 Mab and the ploidy status (*P* = 0.0289, *r*s = −0.4658; Spearman test) but not, when the percentage of cells showing 14F7 Mab staining was evaluated (Tables 4 and 5). When the intensity of reaction of 14F7 Mab in tumors with diploid and aneuploid status was compared, a statistically significant difference was observed (*P* = 0.0176, Chi-square test). In aneuploid tumors, no correlation with the S-phase fraction (SPF) was observed. Nevertheless, in diploid tumors, the intensity of reaction of 14F7 Mab correlate with the SPF (*P* = 0.0208, *r*s = 0.5126; Spearman test). Diploid tumors with higher SPF were mostly recognized by 14F7 Mab. However, no correlation was obtained when the percentage of 14F7 Mab positive cells was studied. Interestingly, aneuploid tumors displayed higher S-phase fraction than diploid ones (*P* = 0.0010, Chi-square test). By the contrary, lung tumors with higher mitotic index showed a reduction in the intensity of reaction with 14F7 Mab (*P* = 0.0284; *r*s = −0.4143; Spearman test) (Table 6). No correlation was observed when the percentage of positive cells was analyzed (Table 4). 

### 3.5. Overall Survival of Patient Was Strongly Associated to the Intensity of Reaction with 14F7 Mab, but Not with Ploidy

**Figure 4:**
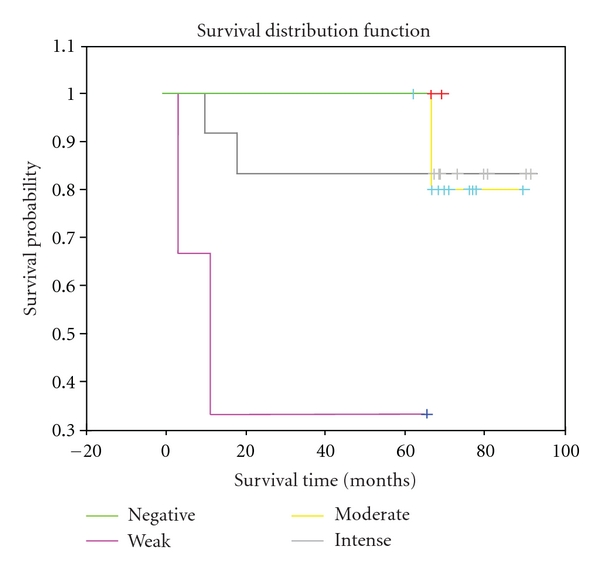
Kaplan-Meier estimate of overall survival among NSCLC patients showing different intensities of reaction with 14F7 Mab (*P* = 0.0125, Log rank test).

**Figure 5:**
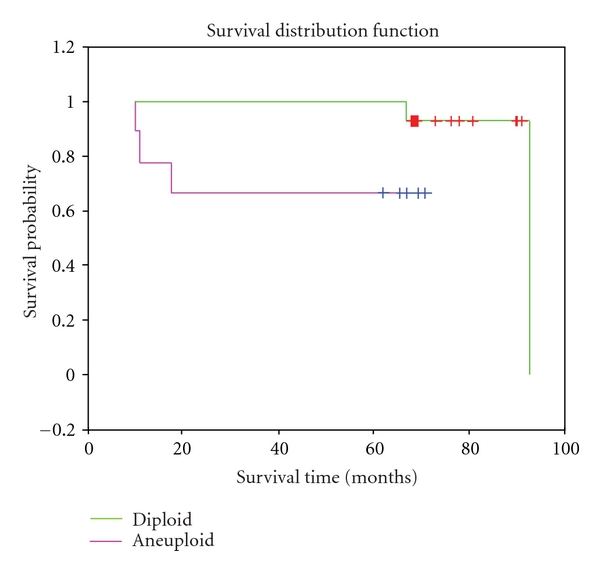
Kaplan-Meier estimate of overall survival among NSCLC patients showing diploid and aneuploid DNA-content status (*P* = 0.0692; Log rank test).

The intensity of 14F7 Mab was strongly associated with overall survival of patient (*P* = 0.0125, Log rank test). Statistical significant differences were detected when the weak intensity of reaction was compared with moderate and intense patterns (*P* = 0.0028 and *P* = 0.0423; Log rank test, resp.). However, no difference was observed between moderate and intense patterns of intensity (*P* = 0.9824; Log rank test) (Figure 4). Ploidy was not found to be associated with the overall survival of NSCLC patient (*P* = 0.0692; Log rank test) (Figure 5). 

### 3.6. The 14F7 Mab Immunorecognition Was Also Observed in Nasopharyngeal Carcinomas

**Figure 6:**
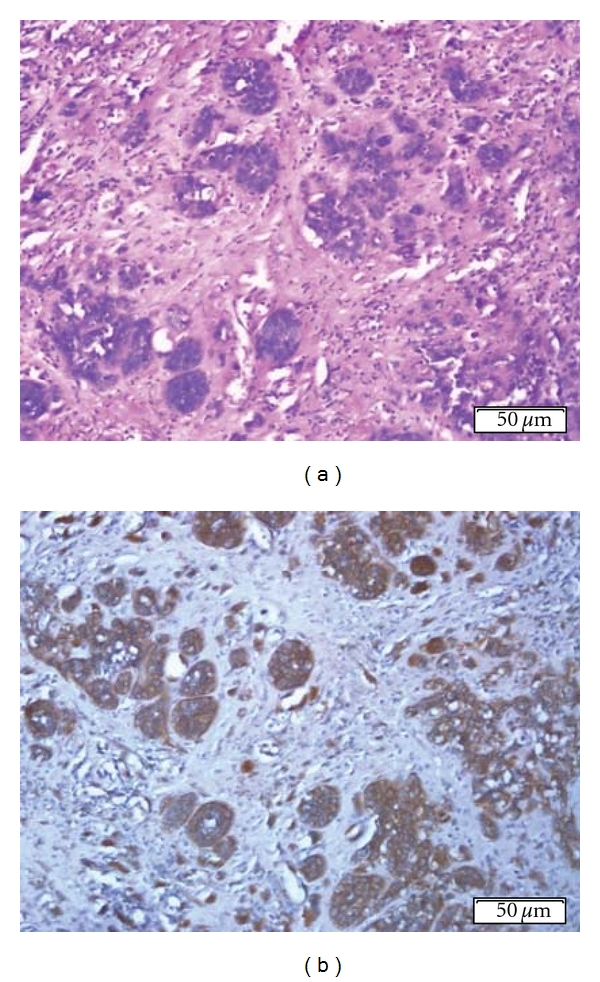
Hematoxylin and eosin staining of poorly differentiated nasopharyngeal carcinoma (a). Note: a strong and finely granular immunoreactivity of 14F7 Mab mainly located in cell membrane of malignant cells (b). Black bar = 50 *μ*m.

Normal tissues (0/2) were not stained with 14F7 Mab. However, a weak immunorecognition of 14F7 Mab was detected in more than 50% of nasopharyngeal dysplastic cells (1/1). In addition, a weak-to-moderate reaction was observed in *in situ* carcinoma (2/2). Concerning to nasopharyngeal carcinoma, 13/14 (92.8%) of them were recognized by 14F7 Mab not taking into account the grade of differentiation. The reaction was located mainly on the plasmatic membrane of malignant epithelial cells, although a cytoplasmatic pattern was evidenced (Table 7, Figure 6). No statistically significant difference was evidenced when the intense of reaction of 14F7 Mab in lung squamous cell carcinoma and nasopharyngeal carcinoma were compared (*P* = 0.5743, Chi-square test). 

## 4. Discussion

Up to date, TNM staging after surgery is considered the most important prognostic factor in patients with NSCLC [16, 17]. However, the pathology-based TNM stage classification has been considered to provide imprecise information about the survival rates [18]. In consequence, numerous studies are currently focusing in the evaluation of other biological and molecular prognostic factors [3, 19]. 

In this study, we show the immunoreactivity of 14F7 Mab, an IgG1 highly specific raised against N-glycolyl GM3 ganglioside, in almost all NSCLC sections, not depending on age, gender, tumor size, tumor stage, histopathological classification, grade of differentiation, and necrosis index. The patter of staining was finely granular and was localized in both cell membrane and cytoplasm of malignant epithelial cells as it was previously described [13, 14, 20, 21]. 

The role of NeuGcGM3 ganglioside in tumoral progression as well as its suppressor properties has been previously reported [22, 23]. The expression of NeuGcGM3 has been considered responsible of tumor-induced dendritic cells (DCs) suppression in NSCLC patient. NeuGcGM3 inhibit DCs differentiation, maturation, and migration based in the inverse correlation observed between NeuGcGM3 expression and infiltrating mature dendritic cells. Conversely, the expression of this ganglioside has been associated with a favorable survival in NSCLC patients, suggesting its prognostic value [13]. Here, we observed a strong association between the higher levels of the 14F7 Mab immunorecognition and increased overall survival rates. Patients with a low intensity of reaction with 14F7 Mab showed a poorer overall survival as compared with those that were intense recognized by 14F7 Mab. Our results are in agreement with those described by van Cruijsen et al. concerning to a positive prognostic factor of 14F7 Mab staining in NSCLC patient [13]. However, NeuGcGM3 expression was associated with a more aggressive biological behavior in neuroectodermal tumors [24]. 

Both the proliferative activity of tumor cells and mitosis count has been considered as potential prognostic factors in lung cancer [25, 26]. Here, we found a positive association between the intensity of reaction of 14F7 Mab and the proliferation index (S-phase fraction) in diploid NSCLC but not in aneuploid tumors, measured by flow cytometry technology. Nevertheless, the mitotic index observed in these samples showed a negative association with the 14F7 Mab staining. The effects of the N-acetyl variant of GM3 ganglioside in the cell cycle have been previously reported. GM3 inhibit cancer cell proliferation by modulating the expression of cell cycle regulation proteins. GM3 dramatically increases cyclin-dependent kinase (CDK) inhibitor p21^WAF1^ expression through the accumulation of p53 protein by the PTEN-mediated inhibition of the PI-3K/AKT [27]. PTEN also resulted in the inhibition of cell cycle progression through the CDK inhibitor p27^kip1^ [28]. Previously, no significant correlation between the percentage of positive cells for the Ki-67 proliferating antigen and the expression of NeuGcGM3 was detected [24]. Although, it is known that the nuclear antigen recognized by Ki-67 antibody provides a reliable method for evaluating tumor growth fraction in lung cancer, it is expressed during the G_1_, S, G_2_, and M phases of the cell cycle [29] making impossible to distinguish between S-phase and mitosis. However, the relation between p53 oncoprotein and NeuGcGM3 ganglioside expressions have been previously published [30]. These results permit us to consider a possible cell cycle inhibitory effect of the antigen recognized by 14F7 Mab in malignant cells, probably similar to that induced by GM3. Interestingly, the only structural difference between N-acetyl and N-glycolyl neuraminic acid is a single-oxygen atom at the C-5 position of NeuGc. Therefore, experiments looking for the possible relation between the 14F7 Mab recognition and the expression of PTEN, p53, and CDK proteins are ongoing in our lab. 

On the other hand, investigations regarding the prognostic value of ploidy have been greatly facilitated by the application of flow cytometry using nuclei isolated from paraffin-embedded tissues [26]. DNA-content (ploidy) abnormalities have been considered as a heterogeneous spectrum of impaired tumor cell DNA histogram patterns. The p53 tumor suppressor gene involved in the arrest of cell growth is also related with DNA repair in response to DNA damage [31]. In our study, the intensity of reaction with 14F7 Mab showed statistical significant differences when diploid and aneuploid NSCLC were compared. Tumors with diploid status were mostly reactive with 14F7 Mab, while aneuploid samples displayed higher levels of S-phase fraction. Curiously, tumors with increased 14F7 Mab intensity of reaction also exhibited a decreased in the mitotic index. 

A highly significant relationship between ploidy and Ki-67 has been previously reported [32]. In a similar study, Pence et al. as well as, Ludovini et al. found a higher Ki-67 immunostaining in aneuploid tumors; however, no association between DNA content and Ki-67 expression was detected [29, 33]. In addition, we found a trend for an association between aneuploidy status and poor overall survival in NSCLC, although no significant difference was obtained. However, the disturbed cellular DNA-content resulting from increased genomic instability have been associated with poor prognosis in lung carcinomas, the negative effects of aneuploidy in the overall survival of patient with NSCLC still remain controversial [29, 34]. 

Tumor necrosis is considered a consequence of chronic cellular hypoxia and has been considered an adverse risk factor for survival and recurrence in patients with NSCLC [35] leading to a more aggressive tumour phenotype and to a poor prognosis [36]. It is known that malignant cells display some genetic changes that permit them to survive and even proliferate in a hypoxic environment. Tumor hypoxia induces the transcription in the cells of a sialic acid transporter facilitating the incorporation of NeuGc molecule from the external medium to the gangliosides [37]. In this study, no correlation was observed when the 14F7 Mab reactivity and the necrosis index were compared. However, the expression of NeuGc-containing gangliosides (e.g., NeuGcGM2) in some tumors has been associated with tumor hypoxia [37]. 

As it was previously described, gangliosides are components of eukaryotic cell membranes [38], structure intimately related to cell morphology [39]. While GM3 is a normal constituent of the plasmatic membrane of the majority of human cells [40], the difference between N-acetyl and N-glycolyl neuraminic acid [41] has converted NeuGcGM3 in a tumor-associated antigen [42]. Here we found a relation between the number of 14F7-Mab-positive cells and the degree of cell pleomorphism in NSCLC sections. The reactivity of this Mab was evidenced in cell membrane and also in the cytoplasm of the malignant cells. The decreasing synthesis of ganglioside GM3 has been associated with a variety of changes in a number of processes that involve the actin cytoskeleton [43]. Interestingly, the primary intracellular determinant of cell morphology is precisely the cytoskeleton [39]. Previous reports have suggested a strong connection between both N-acetyl [44] and N-Glycolyl [45] function of GM3 gangliosides and actin filaments. Aberrant expression of the antigen recognized by 14F7 Mab seems to be in relationship with some changes in the morphology of the malignant cells. 

Nasopharyngeal carcinoma (NPC) is a squamous cell carcinoma that commonly affects certain geographic areas, such as southern Asia. A variety of molecules have been evaluated in NPC in order to consider them as potential target for novel treatments due to a number of molecular abnormalities characteristic of NPC [46]. Almost all NPCs at this serie were recognized by 14F7 Mab not taking into account the grade of differentiation, similar to those obtained in lung squamous cell carcinomas. To our knowledge, it is the first report in the literature about the 14F7 Mab staining in NPC. The expression of other N-glycolyl neuraminic acid containing glycoconjugate on the cell surface of NPC sections using membrane immunofluorescence staining with chicken antiserum against N-glycolylneuraminyl- lactosylceramide (HD3) has been previously reported [6]. Curiously, some alterations in the cellular proliferation pathways such as the AKT pathway and mitogen-activated protein kinases joint to aberrations in cell cycle involving factors such as p16 and CDKs have been found in NPC [46]. Experiments looking forward the possible prognostic factor of 14F7 Mab in NPC immunostaining should be designed by our group. 

Encouraging results have been obtained in NSCLC patient using molecular cancer vaccines. Hernández et al. reported the immune response against NeuGcGM3 in NSCLC patients treated with 1E10 (an anti-idiotypic Mab that mimicry NeuGcGM3). Patient immune sera were able to induce complement-independent cell death of NeuGcGM3-expressing X63 murine myeloma target cells [4]. Interestingly, the ability of 14F7 Mab to directly kill the NeuGcGM3-positive murine myeloma cells without participation of complement have been previously reported [45, 47]. Moreover, the passive treatment with 14F7 Mab produces a strong suppression of tumor growth, similar to the antitumoral response obtained with standard chemotherapy treatment [47]. Fortunately, patients that developed an immune response against NeuGcGM3 showed longer median survival times [4]. 

In summary, we are showing the immunoreactivity of 14F7 Mab, a highly specific IgG1 raised against NeuGcGM3 in a serie of non-small-cell lung carcinoma as well as in nasopharyngeal carcinoma. The relation between the intensity of reaction with 14F7 Mab and a higher overall survival rates in diploid NSCLC joint to a decreased mitotic index in these patients permit us to support the antigen recognized by 14F7 Mab as a good prognostic factor in these patients. Additionally, we described differences in the intensity of reaction with 14F7 Mab between diploid and aneuploid carcinomas, although we could not establish differences in the overall survival between diploid and aneuploid tumors. Our data also support the continuous use of anti-NeuGc therapy in NSCLC patient due to a higher reactivity observed with 14F7 Mab. Experiments looking for a better understating of the possible mechanisms involved in these results are ongoing in our group. Simultaneously, a phase III clinical trial in NSCLC patient using the 1E10 molecular cancer vaccine had started in our country. In addition, experiments looking for a chimerical and/or humanized counterpart of the murine 14F7 Mab as well as their evaluation are in progress.

## 5. Conclusions

The high expression of NeuGcGM3 as recognized by 14F7 Mab in NSCLC as well as in nasopharyngeal carcinoma, but not in sections, supports the continuous use of this molecule as target in the immunotherapy of NSCLC patient and opens up this possibility in nasopharyngeal carcinoma. The staining with 14F7 Mab in NSCLC is in relationship with higher overall survival of patients, constituting a positive prognostic factor.

## Figures and Tables

**Table 1:** Patient characteristic and pathological features.

Features	No. (%)
Gender	
Female	9/32 (28.1)
Male	23/32 (71.9)
Age (years)	
<60	21/32 (65.7)
60–70	6/32 (18.7)
>70	5/32 (15.6)
Tumor size (cm)	
<3	6/32 (18.7)
>3	26/32 (81.3)
Tumor stage	
I	16/32 (50.0)
II	7/32 (21.9)
III A	2/32 (6.3)
III B	1/32 (3.1)
IV	6/32 (18.7)
Histopathological type	
Squamous cell carcinoma	12/32 (37.5)
Adenocarcinoma	9/32 (28.1)
Large cell carcinoma	9/32 (28.1)
Other	2/32 (6.3)
Grade of differentiation	
Well	3/32 (9.4)
Moderate	13/32 (40.6)
Poor	14/32 (43.7)
Undifferentiated	2/32 (6.3)
Degree of cell pleomorphism	
No evident	3/32 (9.4)
Low	10/32 (31.2)
Moderate	11/32 (34.4)
High	8/32 (25.0)
Necrosis index	
No evident	9/32 (28.1)
<50%	12/32 (37.5)
>50%	11/32 (34.4)
Mitotic index	
No evident	2/32 (6.3)
Low	10/32 (31.2)
Moderate	15/32 (46.9)
High	5/32 (15.6)

No. Number of cases, % percentages.

**Table 2:** Immunoreactivity of the 14F7 Mab in normal tissue and lung carcinoma samples.

Histopathological classification	14F7 Mab reactivity		
	No. (%)	Intensity range	% of Positive cells
Normal tissues	0/4 (0)	−	0
Non-small-cell lung cancer	26/28 (92.9%)		
Squamous cell carcinoma	10/10 (100)	+/+++	1–3
Adenocarcinoma	8/9 (88.8)	++/+++	2/3
Large cell carcinoma	6/7 (85.7)	+/+++	2/3
Others	2/2 (100)	++/+++	1/3
Small cell carcinoma	3/3 (100)	+/+++	2/3

Intensity: − negative, + weak, ++ moderate, +++ intense. Positive cells: 0 (negative to less than 5%), 1 (6–25%), 2 (26–50%) and 3 (more than 50%).

**Table 3:** Correlation between pathological features and 14F7 Mab immunorecognition in non-small-cell lung carcinoma.

Pathological features	14F7 Mab reactivity		
	No.	Intensity of reaction	% of positive cells
		*P* value	*P* value
Gender	28	0.1723	0.1152
Age	28	0.8443	0.6818
Tumor size	28	0.7052	0.8801
Tumor stage	28	0.3797	0.0760
Type of carcinoma	28	0.7786	0.9090
Grade of differentiation	28	0.3155	0.3192
Degree of pleomorphism	28	0.0713	0.0001
Necrotic index	28	0.2021	0.1804

No. Number of cases, % percentages, *P* value using Spearman correlation test.

**Table 4:** Correlation between proliferation features and 14F7 Mab staining in non-small-cell lung carcinoma.

Proliferation features	14F7 Mab reactivity		
	No.	Intensity of reaction	% of positive cells
		*P* value	*P* value
DNA content (ploidy)	22	0.0289	0.3065
S-phase fraction (SPF) diploid	20	0.0208	0.2319
S-phase fraction (SPF) aneuploid	20	0.0651	0.4226
Mitotic index	28	0.0284	0.3147

No. Number of cases, % percentages, *P* value using Spearman correlation test.

**Table 5:** Relation between DNA content (ploidy) and 14F7 Mab reactivity in non-small-cell lung carcinoma.

14F7 Mab reactivity	Ploidy			
	Diploid	Aneuploid	Spearman	
	No. (%)	No. (%)	*P* value	*r*s
Intensity of reaction			0.0289	−0.4658
Negative	0/11 (0)	2/9 (22.2)		
Weak	0/11 (0)	2/9 (22.2)		
Moderate	3/11(27.3)	2/9 (22.2)		
Intense	8/11 (72.7)	3/9 (33.3)		
Total	**11/11 (100)**	**7/9 (77.7)**		

No. Number of cases, % percentages, *P* value using Spearman correlation test, *r*s Spearman correlation coefficient.

**Table 6:** Relation between S-phase fraction and mitotic index with the 14F7 Mab immunoreactivity in diploid non-small-cell lung carcinoma.

Proliferation rates	14F7 Mab reactivity					
	Negative	Weak	Moderate	Intense	Spearman correlation	
	No. (%)	No. (%)	No. (%)	No. (%)	*P* value	*r*s
S-phase fraction (SPF)					0.0208	0.5126
0–5	—	—	—	—		
6–25	—	3 (27.3)	—	—		
26–50	—	—	6 (54.5)	—		
>50	—	—	—	2 (18.2)		
Total	**0**	**3 (27.3)**	**6 (54.5)**	**2 (18.2)**		
Mitotic index					0.0284	−0.4143
No evident	—	—	—	2 (7.1)		
Low	1 (3.6)	—	2 (7.1)	6 (21.4)		
Moderate	1 (3.6)	2 (7.1)	7 (25.0)	3 (10.7)		
High	—	1 (3.6)	2 (7.1)	1 (3.6)		
Total	**2 (7.1)**	**3 (10.7)**	**11 (39.3)**	**12 (42.8)**		

No. Number of cases, % percentages, *P* value using Spearman correlation test, *r*s Spearman correlation coefficient.

**Table 7:** Immunorecognition of 14F7 Mab in nontumoral and nasopharyngeal carcinoma.

Histopathological classification	14F7 Mab reactivity		
	No. of cases (%)	Intensity range	% of positive cells
Nontumoral tissues			
Normal	0/2 (0)	−	−
Dysplasia	1/1 (100)	+	3
Squamous cell carcinoma (SCC)			
*In situ *	2/2 (100)	+/++	1/3
Well	4/4 (100)	++/+++	1–3
Moderately	3/3 (100)	+/+++	1–3
Poorly	5/6 (83.3)	+/++/+++	1–3
Undifferentiated	1/1 (100)	+++	3
Total (SCC)	**13/14 (92.8)**		

Intensity: − negative, + weak, ++ moderate, +++ intense. Positive cells: 0 (negative to less than 5%), 1 (6–25%), 2 (26–50%) and 3 (more than 50%).
